# 5-Aminolevulinic Acid Guided Sampling of Glioblastoma Microenvironments Identifies Pro-Survival Signaling at Infiltrative Margins

**DOI:** 10.1038/s41598-017-15849-w

**Published:** 2017-11-15

**Authors:** James L. Ross, Lee A. D. Cooper, Jun Kong, David Gutman, Merete Williams, Carol Tucker-Burden, Myles R. McCrary, Alexandros Bouras, Milota Kaluzova, William D. Dunn, Duc Duong, Constantinos G. Hadjipanayis, Daniel J. Brat

**Affiliations:** 1 Departments of Pathology and Laboratory Medicine, Emory University, Atlanta, GA 30322 Georgia; 2 Biomedical Informatics, Emory University, Atlanta, GA 30322 Georgia; 3 Neurology, Emory University, Atlanta, GA 30322 Georgia; 4 Pediatrics, Emory University, Atlanta, GA 30322 Georgia; 5 Winship Cancer Institute, Emory University, Atlanta, GA 30322 Georgia; 6 Emory University School of Medicine, Emory University, Atlanta, GA 30322 Georgia; 7 Emory University Graduate Program in Cancer Biology, Atlanta, GA 30322 Georgia; 8 Biomedical Engineering, Emory University/Georgia Institute of Technology, Atlanta, GA 30322 Georgia; 9 Department of Neurosurgery, Icahn School of Medicine at Mount Sinai, New York, NY 10003 USA

## Abstract

Glioblastoma (GBM) contains diverse microenvironments with uneven distributions of oncogenic alterations and signaling networks. The diffusely infiltrative properties of GBM result in residual tumor at neurosurgical resection margins, representing the source of relapse in nearly all cases and suggesting that therapeutic efforts should be focused there. To identify signaling networks and potential druggable targets across tumor microenvironments (TMEs), we utilized 5-ALA fluorescence-guided neurosurgical resection and sampling, followed by proteomic analysis of specific TMEs. Reverse phase protein array (RPPA) was performed on 205 proteins isolated from the tumor margin, tumor bulk, and perinecrotic regions of 13 previously untreated, clinically-annotated and genetically-defined high grade gliomas. Differential protein and pathway signatures were established and then validated using western blotting, immunohistochemistry, and comparable TCGA RPPA datasets. We identified 37 proteins differentially expressed across high-grade glioma TMEs. We demonstrate that tumor margins were characterized by pro-survival and anti-apoptotic proteins, whereas perinecrotic regions were enriched for pro-coagulant and DNA damage response proteins. In both our patient cohort and TCGA cases, the data suggest that TMEs possess distinct protein expression profiles that are biologically and therapeutically relevant.

## Introduction

Glioblastoma (GBM) is a highly aggressive primary brain tumor characterized by dismal patient outcomes, with median overall survival of 11–15 months^1^. Despite maximal safe resection, adjuvant chemotherapy and radiation, nearly all patients eventually relapse. The degree of maximal tumor resection is correlated with patient prognosis, yet distinguishing between neoplastic and healthy tissue at resection margins remains a challenge, and the diffusely infiltrative nature of the disease precludes complete resection^2,3^. In almost all cases, GBM recurrence occurs near the site of previous resection, suggesting that therapeutic targeting efforts should focus on this region^4,5^.

GBM has three dominant tumor microenvironments (TMEs) that include the perinecrotic region (PN), bulk tumor (BT), and the infiltrative tumor margin (TM). The perinecrotic zone is characterized by severe hypoxia and contains a hypercellular zone of pseudopalisading cells that are enriched for glioma stem cells^6,7^. Bulk tumor is characterized by densely packed sheets of infiltrating glioma cells with high mitotic activity, while tumor margins are considered the intersection of infiltrating tumor and normal brain. Throughout this micro-environmental diversity of GBM, there is an uneven distribution of oncogenic events and signaling networks^8–13^. While diagnostic testing and most molecular research is typically performed on bulk-resected tissue, the most clinically and therapeutically relevant neoplastic tissue remains in the patient’s brain at the tumor margin. Precise targeting of underlying molecular characteristics present at the margins would be a strategy to improve clinical outcomes^14^. To identify signaling networks and potential druggable protein targets across TMEs and specifically at tumor margins, we utilized fluorescence-guided surgery after 5-aminolevulinic acid (5-ALA) administration, followed by proteomic analysis of precisely defined TMEs.

5-ALA is a newly FDA approved^15^ pro-agent that is metabolized to the fluorescent metabolite protoporphyrin IX (PpIX) in malignant glioma cells^16^. It is orally administered and bioavailable to the brain, where its incomplete metabolism by glioma cells leads to the selective accumulation of PpIX, which is excitable with ultraviolet blue light (λ = 400–410 nm) and fluoresces red-violet (635 nm and 704 nm)^17^. PpIX fluorescence permits real-time visualization and localization of tumor tissue for fluorescence-guided surgery and a greater extent of resection. Previous studies, including our own, have demonstrated that red-violet fluorescence is absent in the region of central necrosis; that areas of strong 5-ALA fluorescence intensity and intraoperative spectroscopic signal correspond to high-density, proliferating BT cells; and that weak fluorescence intensity and intraoperative spectroscopic signal correspond to infiltrating TM tissue. These features allow for the real-time intraoperative distinction of PN, BT, and TM tissue^16,18–23^.

**Figure 1:**
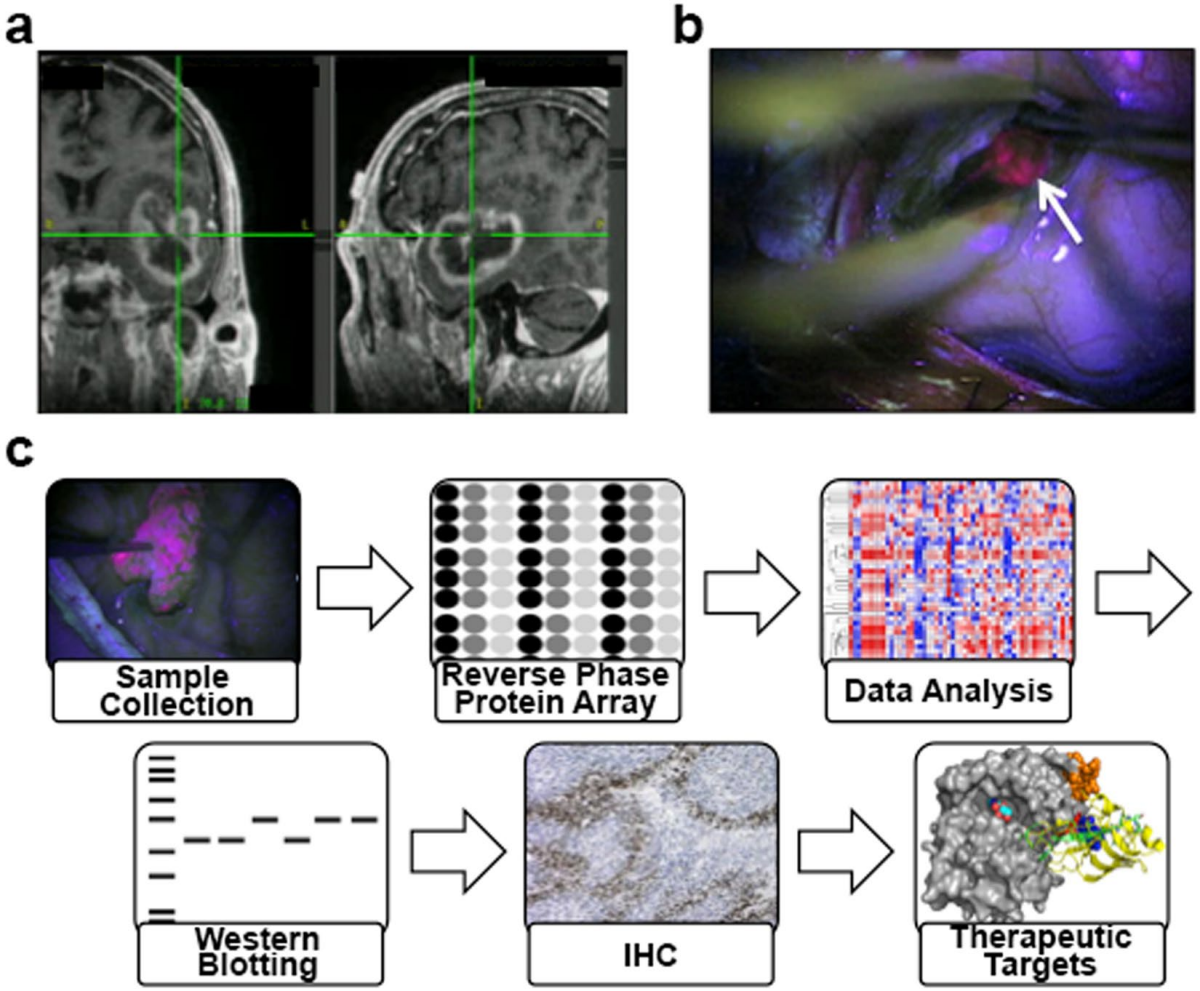
Study workflow. **(a)** T1-weighted MRI stealth scan showing a contrast-enhancing GBM with localization during surgery. **(b)** Fluorescent view of surgical field during resection. White arrow points to fluorescent tumor bulk tissue after accumulation of protoporphyrin IX (PpIX) and excitation at λ = 400–410 nm using ultraviolet blue light. **(c)** Study workflow. Tumor microenvironments were distinguished by the presence or absence of PpIX fluorescence due to 5-ALA administration. Protein was isolated from PN, BT, and TM tissue samples and Reverse Phase Protein Array (RPPA) was performed. Data was then processed and oncogenic proteins were chosen for validation studies and immunohistochemistry (IHC).

In our studies, thirteen patients with high-grade astrocytomas were orally administered 5-ALA before tumor resection and the three tumor regions (PN, BT, TM) were defined and sampled intraoperatively (Fig. 1a–c). Reverse phase protein array (RPPA) was then performed on the samples using a set of 205 antibodies against common oncogenic proteins in order to detect differential expression patterns across the TMEs. Here we report on the differentially expressed proteins and signaling networks across the GBM TMEs, including the pro-survival and anti-apoptotic networks at the infiltrative margins that could represent targets of therapy.

## Results

### Patient Clinical Data Set

This study included 13 previously untreated patients with high-grade astrocytomas, either anaplastic astrocytoma (AA; n = 3, WHO grade III), or glioblastoma (GBM; n = 10, WHO grade IV), who were administered 5-ALA for FGS and tumor sampling and resection. There were 5 males and 8 females, ranging in age from 29–75 years (mean = 56 years)(Table 1). Five tumors were *EGFR* amplified, all of which were *IDH* wild type GBMs. Ten tumors showed *PTEN* loss (1 Grade III; 9 Grade IV), and all tumors that were *EGFR* amplified were also *PTEN* deleted. Five tumors showed *MGMT* promoter methylation, all of which were GBM. Three tumors harbored *IDH-1* mutations (2 Grade III; 1 Grade IV).

**Table 1:** Clinical Characteristics of Patient Cohort.

Clinical Characteristics of the Patient Sample Set					
Characteristic	Total (N = 13)	EGFR Amplification (N = 5)	PTEN Loss (N = 10)	MGMT Methylation (N = 5)	IDH1 Mutation Positive (N = 3)
Histologic type and grade					
Grade III (AA)	3	0	1	0	2
Grade IV (GBM)	10	5	9	5	1
Age at diagnosis (yrs)					
Mean	56.3 ± 14.6	65 ± 7.4	60.6 ± 12.2	66.8 ± 5.5	41 ± 19.1
Range	29–75	56–75	31–75	60–75	29–63
Male sex	5	2	4	2	2
Female sex	8	3	6	3	1
Tumor location					
Frontal lobe	2	2	2	1	0
Parietal lobe	4	1	3	1	1
Temporal lobe	7	2	5	3	2
Laterality					
Left	7	3	5	4	1
Midline	0	0	0	0	0
Right	6	2	5	1	2

Diagnostic and molecular characteristics of the patient cohort used for this study are displayed.

### GBM Tumor Microenvironments Have Distinct Protein Expression Patterns

**Figure 2:**
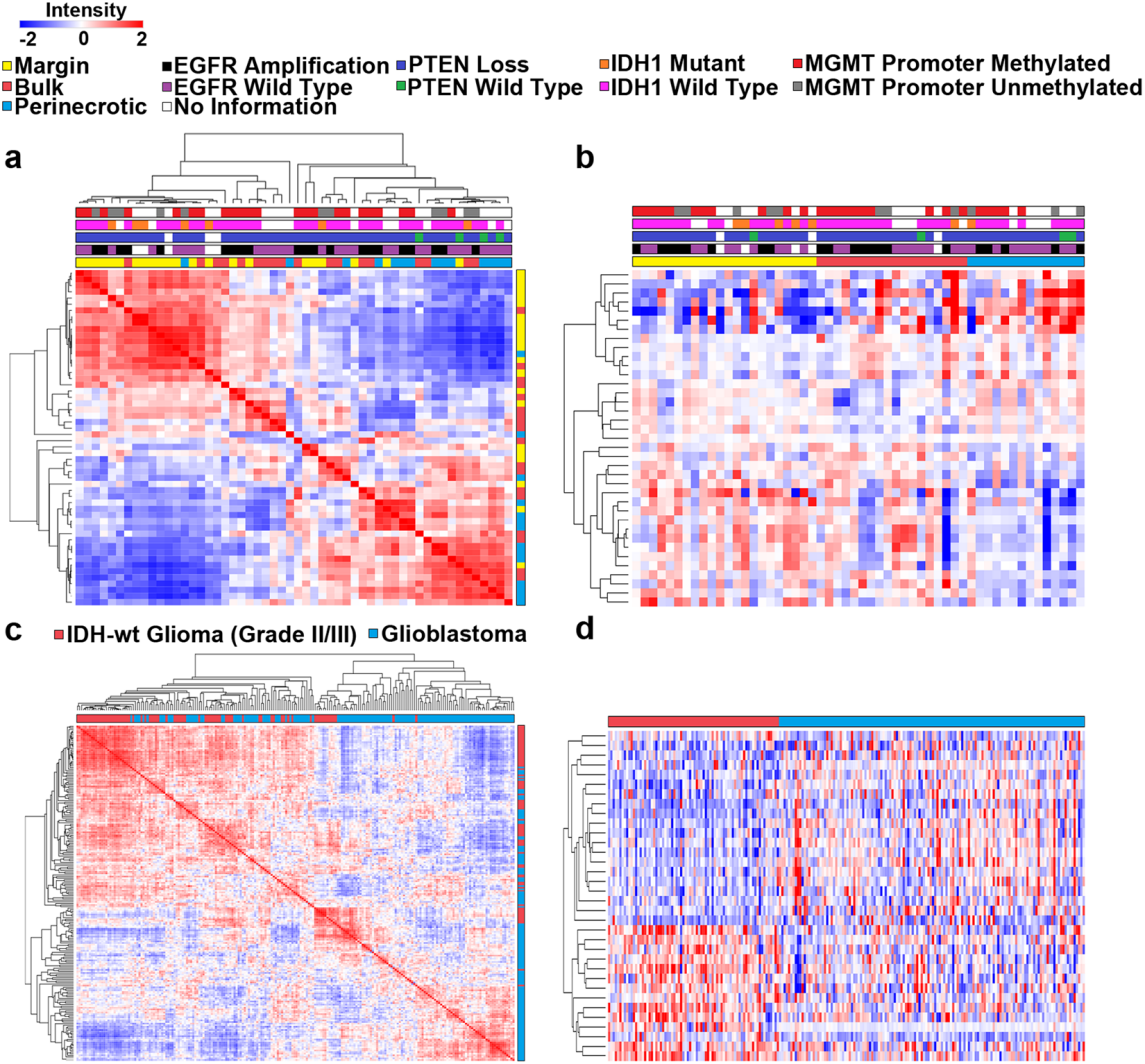
Heat maps of protein expression for our dataset and for the TCGA GBM and IDH-wt grade II/III glioma dataset. **(a)** Similarity matrix of the 54 tumor samples described by the 37 differentially expressed proteins (p < 0.05). Molecular profiling of each sample is also provided. **(b)** Supervised hierarchical clustering of the 37 differentially expressed proteins (p < 0.05). Samples are supervised by tumor microenvironment as columns and clustered by protein expression with three clusters present. **(c)** Similarity matrix of the 212 untreated tumors in the TCGA RPPA database including IDH-wt grade II/III glioma (n = 76) and GBM (n = 136) described by the 34 differentially expressed proteins (p < 0.01). **(d)** Supervised hierarchical clustering of the 34 differentially expressed proteins (p < 0.01) among IDH-wt grade II/III gliomas and GBMs. Samples are supervised by tumor grade as columns and clustered by protein expression with two main clusters present.

**Figure 3:**
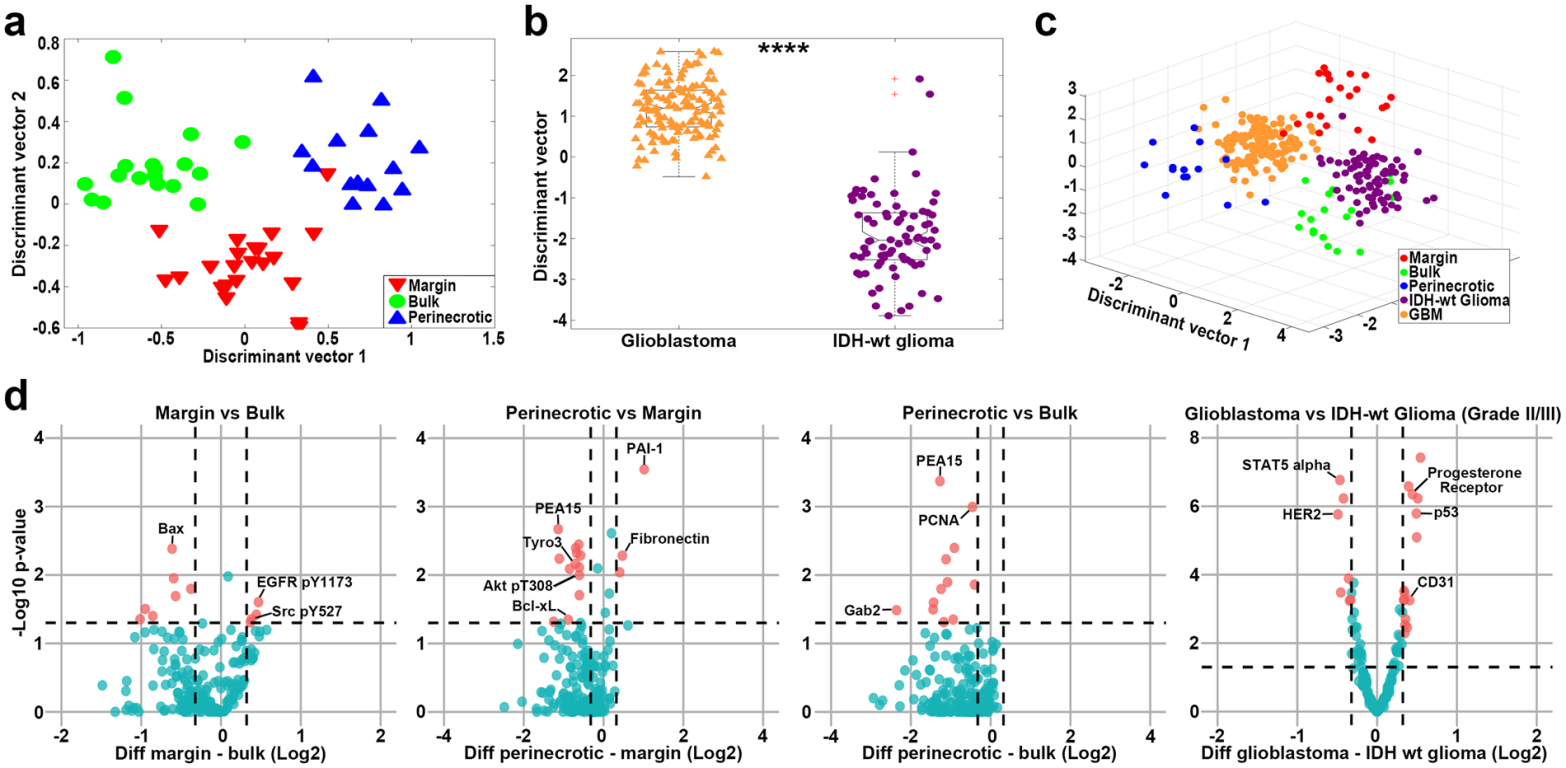
Characterization of individual tumor regions using linear discriminant analysis and volcano plots. **(a)** Linear Discriminant Analysis mapping the differentially expressed proteins for each sample in our dataset into a 2-dimensional subspace, demonstrating region separability. **(b)** Linear Discriminant Analysis on the TCGA GBM and IDH-wt grade II/III glioma dataset, demonstrating strong grade-associated separation (two-sample t-test p = 1.12e-44). **(c)** Linear Discriminant Analysis mapping the 150 shared proteins in both datasets for each sample into a 3-dimensional subspace, demonstrating similarity between PN and GBM samples and BT and IDH-wt grade II/III glioma samples. **(d)** Volcano plots of pairwise microenvironment comparisons (TM-BT, PN-TM, PN-BT, GBM–IDH-wt grade II/III glioma) using protein expression fold change as the x-axis and –Log10 p-value as the y-axis for all proteins detected in the RPPA panels. Thresholds for significance are represented by the horizontal dashed line at 1.3 (−Log10 p-value) and vertical dashed lines at ±0.032 (Log2 fold change). Data points colored in blue do not pass the threshold for consideration as significantly differentially expressed. Red data points pass both thresholds for consideration and represent differentially expressed proteins in the respective tumor region.

Using ANOVA and Tukey-HSD post-hoc statistical tests on the complete RPPA data set that included PN, BT and TM samples, we found that 37 proteins were differentially expressed among the tumor regions (p < 0.05). To determine segregation patterns of proteins represented on the RPPA panel, we created a similarity matrix using the 37 proteins and clustered by sample, which demonstrated that TM samples aggregated together to the greatest degree among the 3 regions (Fig. 2a). PN samples were most clearly separated from the TM, while BT samples were intermingled with both the PN and TM. This trend was evident in unsupervised hierarchical clustering of the 37 differentially expressed proteins as well (Supplemental Fig. 1a), indicating that the GBM TMEs possess distinct regions of protein expression and that PN and TM profiles differ the most. BT displays mixed expression profiles likely due to the heterogeneous nature of GBMs, as well as the spatial relationship between PN and TM regions. To analyze the degree of separability of the three TMEs, we performed linear discriminant analysis (LDA) and mapped the 37 differentially expressed proteins for each sample into a 2-dimensional subspace where class-conditional data are best separated^24^. Using this method, we found that samples from each tumor region were almost exclusively clustered together with little to no overlap with samples from other regions, indicating an even greater difference in TME expression profiles than the similarity matrices would suggest (Fig. 3a).

Using the 37 differentially expressed proteins, supervised clustering by tumor region was then performed in order to define protein and network expression profiles specific to each region and three distinct clustering patterns were observed (Fig. 2b). Cluster one was characterized by low protein expression at the TM, mixed expression in BT, and high expression in PN samples. Of most interest was the significant upregulation of pro-coagulant proteins within the PN, including Plasminogen Activator Inhibitor-1 (PAI-1) and Fibronectin, which both promote thrombosis, a defining feature of GBMs associated with the development of hypoxia and necrosis^6,25^. Other proteins highly upregulated in this cluster included Histone H3, Insulin-Like Growth Factor Binding Protein 2 (IGFBP2), Cyclooxygenase 2 (COX2), Focal Adhesion Kinase (FAK), and Transferrin Receptor (TFRC). Fibronectin and FAK are important mediators of glioma cell migration^26^, and may be a component of hypoxia-induced migration of pseudopalisading cells away from the necrotic core^6^.

The second cluster was characterized by elevated protein expression in the PN region and at the TM. Proteins upregulated in this cluster included DNA damage response proteins MutS Homolog 2 (MSH2), Proliferating Cell Nuclear Antigen (PCNA), and BRCA1 Associated Protein 1 (BAP1). Cyclin E1, which displays copy number gain in 36% of GBMs and whose overexpression results in chromosome instability^27^, was also upregulated in this cluster, as was NF-κB-p65 (pS536), an inflammatory and stress response protein. These results indicate that the PN region specifically expresses a variety of stress response proteins, possibly related to severe hypoxia, acidosis, immune cell infiltrate and tissue damage.

The third cluster was marked by elevated expression at the TM, mixed expression in BT, and low expression in PN samples. This cluster included proteins related to pro-survival processes and the PI3K/Akt/mTOR pathway such as Tyro3, Gab2, p27, Src (pY527), PDK1 (pS241), PTEN, Beta-Catenin, Akt (pT308) and Tuberin. Another interesting protein with high expression at the TM is the N-myc downstream-regulated gene 1 (NDRG1 pT346) which is regulated by HIF-1α, stress and growth signals, and may be a marker of tumor progression^28^. This cluster also includes anti-apoptotic proteins Bcl-XL and PEA-15, which have copy number gains in 39% and 16%, respectively, in GBMs, and are being investigated as potential therapeutic targets for glioma^29,30^. The proteins overrepresented in this cluster suggest that a pro-survival and anti-apoptotic signaling network is present at the TM, likely contributing to the chemotherapy and radiotherapy resistant phenotype observed in GBM. Results were further supported by reactome pathway analysis of differentially expressed proteins, in which the TM showed a marked enrichment of PI3K/Akt and anti-apoptotic signaling pathways while the PN region was enriched in DNA damage and stress response pathways (Supplemental Fig. 2a–c). Although molecular information was available for the tumor samples (*IDH* status, EGFR amplification, PTEN loss, *MGMT* promoter methylation), no discernable expression patterns were evident among these characteristics likely due to small sample sizes. Overall, our results demonstrate that individual proteins and protein networks show differential expression across GBM TMEs.

### TCGA RPPA Data from IDH-Wild Type Glioma and GBM Reveal Distinct Protein Expression Patterns

To compare and potentially validate distinct protein expression patterns across GBM TMEs based on 5-ALA guided sampling, we performed a similar analysis of 76 IDH wild type (wt) WHO grade II/III gliomas and 136 IDH-wt primary GBMs (WHO grade IV) using 176 proteins from the TCGA RPPA database. A full list of analyzed proteins can be found in the supplemental data file. IDH-wt grade II/III gliomas and IDH-wt GBM are genetically similar and differ mostly by the histologic presence of necrosis and microvascular proliferation, which are defining diagnostic criteria of GBM^31,32^. Since TCGA samples of GBM contain perinecrotic as well as bulk tumor, while IDH-wt grade II/III gliomas (by definition) do not contain necrosis, we reasoned that differential protein expression noted between these sample sets would be due mostly to the presence of necrosis (and its associated biological events) and could be used to compare our results from 5-ALA targeted sampling of PN and BT samples. Using the TCGA data, we found that 57 proteins were differentially expressed (p < 0.05) by IDH-wt grade II/III gliomas and GBMs after correction for FDR. In order to determine if protein expression patterns between these two cohorts were distinct from one another we performed LDA on IDH-wt grade II/III gliomas and GBM TCGA samples using these 57 differentially expressed proteins (p < 0.05). We observed strong separation of IDH-wt GBM and IDH-wt grade II/III glioma samples (two-sample t-test p = 1.12e-44), indicating that the protein expression patterns are highly distinctive among tumors that have necrosis and those that don’t (Fig. 3b). To determine if protein expression patterns of IDH-wt GBMs and IDH-wt grade II/III gliomas from the TCGA were similar to PN and BT from our 5-ALA targeted samples, we normalized protein expression values for all samples, and used the 150 proteins that were present in all data sets. We then performed LDA and plotted TM, BT, PN, IDH-wt grade II/III glioma, and GBM expression profiles together in a single 3-D space. We observed that PN samples had most similarity to TCGA GBM samples (Euclidean distance = 2.7707) and that BT samples were most similar to TCGA IDH-wt grade II/III glioma samples (Euclidean distance = 3.4941)(Fig. 3c).

In order to further explore specific protein expression identities and patterns that distinguish TCGA IDH-wt grade II/III glioma from GBM, we plotted the differentially expressed proteins from these datasets using an unsupervised similarity matrix clustered by sample and observed two dominant clusters; one almost exclusively contained GBM samples and the other contained predominantly IDH-wt grade II/III gliomas (Fig. 2c). We then performed supervised hierarchical clustering by tumor class (GBM vs IDH-wt grade II/III gliomas) on this same set of proteins and observed two distinct clusters (Fig. 2d). Cluster one was distinguished by low expression in IDH-wt grade II/III gliomas and high expression in GBMs, although variability among the GBM samples was noted. Proteins in this cluster include hormone receptors Estrogen Receptor alpha and Progesterone Receptor, N-Ras, and Myosin Heavy Chain 11. DNA damage and stress response proteins p53 and Cleaved Caspase 7 were also highly expressed in GBM, reinforcing similar findings of upregulated stress response protein expression in PN samples. Vascular endothelial marker CD31 was highly expressed in GBM samples, reflective of a pro-angiogenic TME. Cluster two was characterized by high expression in IDH-wt grade II/III gliomas and low expression in GBMs. Her2, AMPK-alpha, IGFBP2, and STAT5-alpha were highly expressed in IDH-wt grade II/III gliomas demonstrating the involvement of signaling cascades in these tumors. Pathway analysis also confirmed that PI3K/Akt signaling was enriched in IDH-wt grade II/III glioma samples and apoptotic processes were enriched in GBM samples (Supplemental Fig. 2d). Since we are unaware of the precise site of tumor sampling for TCGA GBM samples, we cannot make definitive comparisons between this data and our own. However, these results demonstrate that protein expression is linked to grade-associated changes, likely highly influenced by the presence of necrosis, supporting our hypothesis that the TME influences protein expression.

### Volcano and Box Plot Analysis

**Figure 4:**
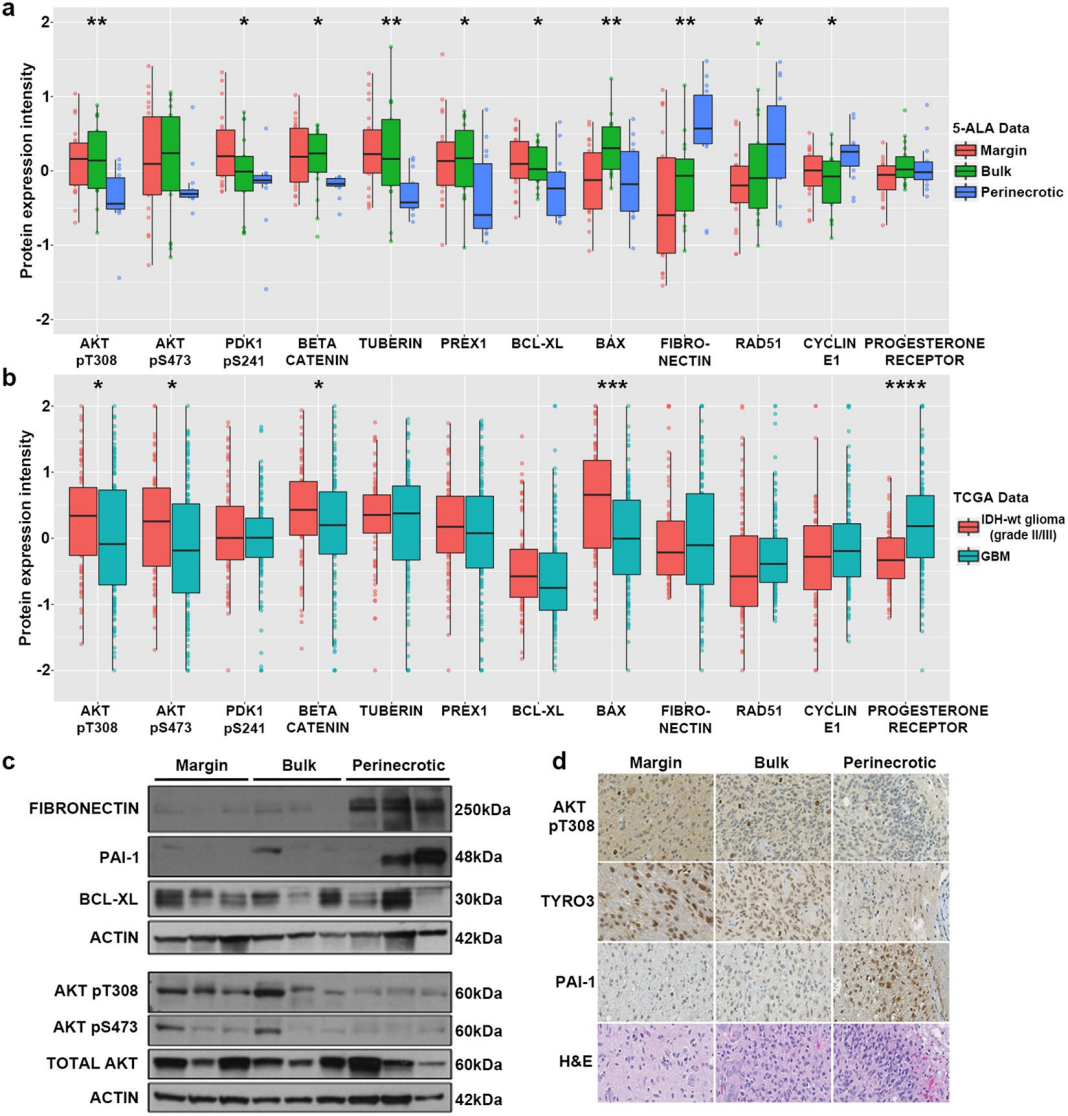
Validation of protein expression using TCGA data, immunohistochemistry, and western blotting. **(a)** Selected box plots of normalized protein expression intensity values for each tumor region (TM, BT, PN) in our dataset **(b)** and in the TCGA dataset (GBM & IDH-wt grade II/III glioma) displaying differential protein expression patterns conserved in both datasets. Asterisk indicates significant difference (*p < 0.05, **p < 0.01, ***p < 0.001, ****p < 0.0001). TM n = 22, BT n = 18, PN n = 14, IDH-wt grade II/III glioma n = 76, GBM n = 136. **(c)** Western blotting for Akt (pT308), Akt (pS473), Fibronectin, Bcl-XL, and PAI-1 using corresponding patient tissue samples. n = 3 patients for each tumor region. Proteins were probed for on two separate blots. Uncropped full length blots can be found in Supplemental Fig. 4. (**d**) Immunohistochemical validation of selected proteins using formalin-fixed, paraffin-embedded GBM tissue samples corresponding to those used in RPPA. Images were acquired at 60x magnification.

In order to identify specific proteins and phospho-proteins that were most differentially expressed across TMEs in our data from 5-ALA guided sampling, volcano and box plots were created after normalization and Log2 transformation. This analysis shows that 13 proteins were overexpressed and 8 proteins underexpressed at the TM; 16 were overexpressed and 4 underexpressed in BT; and 3 overexpressed and 22 underexpressed in PN (Fig. 3d). Anti-apoptotic protein PEA15, favoring expression at the TM, and pro-coagulation protein PAI-1, favoring expression in PN, were the most differentially expressed proteins with respective p-values of 0.00032 and 0.00041. In the TCGA dataset, 27 proteins are overexpressed and 30 proteins underexpressed in IDH-wt grade II/III gliomas compared to IDH-wt GBM. Box plots displayed in Fig. 4a,b represent selected oncogenic proteins that were among the most differentially expressed within specific microenvironments, including Akt (pT308), Akt (pS473), PDK1 (pS241), Beta-Catenin, Tuberin, Prex1, and Bcl-XL. These proteins are all involved in the PI3K/Akt/mTOR or pro-survival pathways and showed overexpression at the TM and under expression in the PN region, representing potential targets for therapy^33^. Fibronectin, Rad51, Cyclin-E1 and Progesterone Receptor are also shown, along with supporting box plots from the IDH-wt grade II/III glioma vs GBM expression data, and demonstrated higher expression in the PN and GBM samples. Using these data, we identified several proteins and pathways with elevated expression in TM samples that could potentially warrant further investigation as therapeutic targets (Table 2). Based on our results, Akt (pT308), Akt (pS473), Tyro3, Fibronectin, PAI-1, and Bcl-XL were chosen for western blot or immunohistochemical validation in patient tissue samples.

**Table 2:** Potential Therapeutic Targets Overexpressed at Tumor Margins.

Potential Therapeutic Targets Overexpressed at Tumor Margins		
Protein	Pathway Involvement	ANOVA p
Akt pT308	PI3K/Akt/mTOR	0.0027
Src pY527	PI3K/Akt/mTOR	0.0038
Tyro3	PI3K/Akt/mTOR	0.0053
PDK1 pS241	PI3K/Akt/mTOR	0.0219
PKC alpha	PI3K/Akt/mTOR, Ras/Raf/MEK	0.0097
PKC alpha pS657	PI3K/Akt/mTOR, Ras/Raf/MEK	0.0059
PKC delta pS664	PI3K/Akt/mTOR, Ras/Raf/MEK	0.0070
NDRG1 pT346	PI3K/Akt/mTOR, Ras/Raf/MEK, Nf-kB,TGFbeta	0.0061
Prex1	Rho/Rac, PI3K/Akt/mTOR	0.0327
Bcl XL	Anti-apoptosis	0.0453
PEA15	Anti-apoptosis	0.0003
Beta Catenin	Wnt/Beta Catenin	0.0255

Proteins that were differentially expressed, with overexpression in tumor margin samples, are displayed along with the signaling pathways they are involved in and their ANOVA p-value.

### Validation of Increased Akt Pathway Activity and Coagulation Factor Expression In Patient Samples

To further validate differential expression among the TMEs, western blotting was performed using protein isolated from patient tissue samples (n = 3 for each tumor region). Akt (pT308), Akt (pS473), PAI-1, Fibronectin, and Bcl-XL were detected and their differential expression patterns confirm our RPPA data (Fig. 4c). There is modest variability noted between patient samples within each tumor region, reflective of sample-to-sample variance, yet expression differences are evident between tumor regions for each protein detected. Both Akt (pT308) and Akt (pS473) were overexpressed in TM samples, with little expression in PN samples, while total Akt showed strong expression in all samples. Additionally, all TM samples expressed anti-apoptotic protein Bcl-XL, supporting the finding that the TM is characterized by overexpression of pro-survival proteins. PAI-1 and Fibronectin were both found to have almost exclusive expression in the PN samples, supporting a pro-coagulant profile in the PN region.

To determine if proteins were expressed specifically in neoplastic cells, we examined the expression of Akt (pT308), Tyro3, and PAI-1 by immunohistochemistry using the corresponding paraffin-embedded patient samples from six cases that contained all three tumor regions. PAI-1 was one of the most differentially expressed proteins among the TMEs, second only to PEA15, while Akt (pT308) ranked fifth (p = 0.00267) and Tyro3, an upstream activator of Akt, ranked eleventh (p = 0.00526). Akt (pT308) and Tyro3 expression levels were higher at the TM and BT than in the PN region, confirming results obtained from our RPPA data, and the expression was highly specific to neoplastic cells, ruling out potential staining of normal brain tissue at the TM. Akt (pT308) showed both cytoplasmic and nuclear expression, with highest cytoplasmic levels at the TM, but with higher nuclear staining in dividing bulk cells (Fig. 4d). Tyro3 also had both cytoplasmic and nuclear expression and was strongest at the TM, with little to no staining in PN regions. PAI-1, whose expression in our dataset and western blotting was lowest at the TM and highest in the PN region, showed greatest expression in the PN regions, specifically in neoplastic GBM cells. Interestingly, PAI-1 staining was strongest in PN areas near medium to large vessels that were occluded by thrombus, specifically in regions directly adjacent to necrosis, suggesting that it may be hypoxia-inducible, involved in thrombosis, or both.

## Discussion

The present study demonstrates that neurosurgical sampling of high-grade glioma guided by differential 5-ALA induced fluorescence is capable of identifying specific TMEs with distinctive protein expression patterns that correlate with the histopathologic features of these regions^19^. This is important, since the oncogenic driving forces of glioma growth that are present centrally in the tumor, near the necrotic core, may be highly distinct from those at the infiltrating edge. While the mechanisms present within the perinecrotic pseudopalisades are biologically interesting, they may not be the most relevant therapeutic targets in a GBM following a gross total resection. Most recurrences occur at or near the resection cavity walls and are due to the regrowth of diffusely infiltrating tumor that could not be completely resected^4,5^. Therefore, differential expression of oncogenic proteins may have therapeutic significance in addition to informing the underlying biology.

One of our chief findings was that the TM is characterized by a pro-survival microenvironment compared to the PN region, with higher expression of anti-apoptotic proteins and proteins involved in the PI3K/Akt/mTOR pathway. A common signaling abnormality of GBM is the substantial upregulation of Akt activity, typically downstream of *EGFR*, *PDGFR* or *c-Met* amplification, *PTEN* loss or activating mutations in *PI3K* subunits, leading to Akt activation in approximately 70% of GBMs^34,35^. Activation of the PI3K/Akt/mTOR pathway leads to chemo-resistance, radio-resistance, tumor cell proliferation, inhibition of apoptosis, and other indirect downstream effects such as increased protein synthesis and induction of angiogenesis via mTOR^36^. The specific targeting of this pathway has been suggested as a viable therapeutic approach and several clinical trials have been aimed at this mechanism^33,34,37^. In our studies, several proteins involved in the PI3K/Akt/mTOR pathway were highly expressed in the BT and at the TM and underexpressed in the PN region. We validated these findings with TCGA data, pathway analysis, western blotting, and immunohistochemical staining for Akt (pT308) and an upstream receptor tyrosine kinase, Tyro3^38,39^. Our results suggest that specific upregulation of these proteins may represent one mechanism by which residual tumor cells at the tumor margin become therapeutically resistant and recur.

While our investigations mostly focused on the TM, we found that the PN region displayed its own biologically interesting upregulation of proteins that are best known for their roles in modulating stress responses and the coagulation cascade. Two of the most significantly overexpressed proteins in the PN region as validated by RPPA data, western blotting, and immunohistochemistry were PAI-1 (*SERPINE1*) and Fibronectin, which both have a direct influence on activation of the coagulation system, the formation of thrombi, and vascular morphogenesis^40^. One of the defining features of GBM is the development of necrosis, severe hypoxia and subsequent hypoxia-induced angiogenesis. It is highly possible that overexpression of PAI-1 and Fibronectin in the PN regions may support a pro-coagulative environment centrally within GBM. PAI-1, a member of the serine protease inhibitor family, inhibits the enzymatic activity of tissue-type plasminogen activator (tPA) and urokinase-type plasminogen activator (uPA) and thus prevents fibrinolysis leading to an increased risk of the development of intravascular thrombosis^41^. More than 80% of GBMs display copy number gains of PAI-1, due to its location on chromosome 7 (7q21.3-q22)(Supplemental Fig. 3a). The expression of PAI-1 and Fibronectin is induced by hypoxia and TGF-β, which could explain upregulation in the PN region, and their expression strengthens resistance to radiation^42,43^. In our study, we observed PAI-1 upregulation by immunohistochemistry specifically near blood vessels that were occluded by thrombus and in cells immediately adjacent to necrosis. We hypothesize that upregulation of PAI-1 and Fibronectin, together with the highly pro-thrombotic protein Tissue Factor, in the central perinecrotic region of GBM may produce a pro-coagulant environment that further supports the development of necrosis and subsequent emergence of distinct TMEs^25^.

In conclusion, we have shown that GBM TMEs are characterized by differential protein expression patterns^19^. Our study demonstrates the utility of 5-ALA as a tool to differentiate TMEs, aiding in the molecular characterization of GBMs. Our results imply that not only does the proteomic landscape depend on the TME but the genetic landscape may be influenced as well, or even responsible for driving these observed differences. This is particularly relevant to current diagnostic and prognostication methods such as molecular subtyping of GBMs and assigning patients to targeted therapies, as the region of sampling may drastically affect characteristic readouts such as chromosomal deletions, receptor tyrosine kinase amplifications, and tumor suppressor mutations and deletions. The findings of differential oncogenic protein expression patterns at the TM should promote further interrogation of potential therapeutic targets and delivery methods that can be specifically directed at this region, since it is most responsible for tumor recurrence^4,5^. As our study was limited to a pre-defined panel of proteins and small sample sizes, future studies should include larger cohorts and unbiased approaches to detect a greater number of targets at the TM. Additionally, further studies are needed to define the influence that stromal cells in the TMEs have on tumor cell protein expression. Overall, our findings have important implications for the use of 5-ALA in tumor resections, molecular classification, and disease management of GBMs.

## Materials and Methods

### Sample Collection

Patients suspected on clinical and neuroimaging evidence to have a primary high-grade glioma, anaplastic astrocytoma (WHO grade III) or GBM (WHO grade IV), were enrolled in the Emory University Institutional Review Board-approved clinical trial, “A Phase 2 Study of 5-Aminolevulinic Acid (ALA) to Enhance Visualization and Resection of Newly Diagnosed or Recurrent Malignant Gliomas,” with written informed consent from all patients to use tissue and clinical data for investigation. All methods in this study were carried out in accordance with relevant guidelines and regulations and all experimental protocols were approved by Emory University. Patients were orally administered 5-ALA (20 mg/kg; Gliolan, Photonamic GmbH Inc, Germany) 3–5 hours prior to neurosurgery^44^. At the time of operation, tumor was visualized based on the presence of red-violet fluorescence following excitation with ultraviolet blue light (λ = 400–410 nm) and a frozen section was used to confirm the diagnosis of a high-grade astrocytoma^3^. Neurosurgical resection was performed with the goal of maximal safe resection using 5-ALA FGS. During the operation, resected tumor was designated as perinecrotic tumor (PN) if it showed fluorescence and was adjacent to the non-fluorescing necrotic center; bulk tumor (BT) if it showed fluorescence and was distant from necrosis and the resection margin; and tumor margin (TM) if the tissue showed diminished fluorescence as it approached the non-fluorescing margin. Tissue samples from each region were frozen in liquid nitrogen for future proteomic studies. Adjacent samples were procured for formalin-fixation and paraffin-embedding for immunohistochemical analysis and histologic review by a neuropathologist. All cases were confirmed as either AA (WHO grade III) or GBM (WHO grade IV) based on final pathological review according to WHO criteria^45^. Diagnostic testing in the hospital lab was performed for *IDH* mutational status, *EGFR* amplification, *PTEN* loss and MGMT promoter methylation status.

### Reverse Phase Protein Array (RPPA)

A total of 54 frozen tissue samples (14 PN, 18 BT, 22 TM) from 13 patients were sent to the University of Texas MD Anderson RPPA core facility in two batches for analysis as previously described^46,47^. Samples were quality controlled for diagnosis and viable tumor cell density over 60%. Detailed reviews of RPPA can be referenced for further details on methodology and its clinical utility^48,49^. The list of all 205 proteins and phospho-proteins that were targeted by antibodies on this array can be found in the supplemental data file.

### Statistical Analysis, Generation of Heat Maps, and Generation of Plots

Each protein was normalized to the mean intensity within the two sample batches. ANOVA and TukeyHSD post-hoc statistical tests^50^ for each pairwise combination (TM-BT, PN-TM, PN-BT) were used to determine differentially expressed proteins of high significance (p < 0.05). Heat maps were generated using the online software Morpheus (http://broadinstitute.org) as hierarchical clusters using the one minus Pearson Correlation metric and average linkage method. Normalized intensity values for each of the 205 proteins, either supervised or unsupervised by tumor region, were used. Box plots were created in R (http://r-project.org) using normalized protein intensities for each tumor region. Volcano plots were created in R using Log2 transformed data after normalization and thresholded for a minimum Tukey-HSD p-value of 0.05 and a minimum fold change of 1.25.

IDH-wt glioma (WHO grade II and III) and GBM (WHO grade IV) RPPA data was downloaded (http://www.cbioportal.org) from the Brain Lower Grade Glioma (TCGA, Provisional) database and the Glioblastoma Multiforme (TCGA, Provisional) database accessed on December 30, 2016. RPPA data was available for investigation on 76 IDH wild type grade II/III gliomas and 136 IDH-wt GBM samples. Samples were normalized by removing all values +/−3sd from the average and then scaling to a maximum 2 and minimum −2. ANOVA was then performed and p-values were corrected using Benjamini-Hochberg for multiple comparisons. To perform linear discriminant analysis we normalized protein expression by z-score individually for each dataset, merged the normalized datasets, and then plotted data using Matlab version R2016b.

### Western Blotting

For validation of differential expression of selected proteins, western blotting was performed on additional frozen aliquots from samples that were included in the RPPA studies. Samples were homogenized and lysed in 1x RIPA buffer including protease and phosphatase inhibitors and then run on a 10% SDS-PAGE gel (BioRAD) and probed with antibodies directed at Akt (CST, 4691 S), Akt pT308 (CST, 2965), Akt pS473 (CST, 3787), PAI-1 (Santa Cruz, sc-5297), Fibronectin (Abcam, ab2413), Bcl-XL (CST, 2764), Actin (Sigma, a5441), and then developed using a secondary HRP conjugated antibody. Two blots were used and when necessary, were stripped with Restore Western Blot Stripping Buffer (Thermo Scientific, 21059) and re-probed with the corresponding antibody.

### Immunohistochemistry

Formalin-fixed, paraffin-embedded GBM tissues corresponding to samples used for RPPA analysis were available for immunohistochemistry (IHC) on 6 cases to validate differentially expressed proteins. Unstained histologic sections cut at 5 microns were deparaffinized and antigen retrieval was performed using 1:10 diluted Target Retrieval Solution (10x) (Dako S1699). Akt pT308 (Abcam, ab38449), Tyro3 (Abcam, ab37839), and PAI-1 (Abcam, ab66705) antibodies were diluted to desired concentrations in Antibody Diluent (Dako S3022) and incubated at room temperature for 40 minutes. Visualization and detection was then established using Rabbit-on-Rodent HRP polymer (Dako K4063) and slides were developed with DAB for 2 minutes. Slides were counterstained using Hematoxylin counterstain (Richard Allan Scientific 7211) diluted 1:5 for 3–5 minutes, dehydrated, cleared in xylene, mounted and coverslipped.

### Data Availability

The TCGA datasets used for analysis can be found at www.cbioportal.com using the Brain Lower Grade Glioma (TCGA, Provisional) database and the Glioblastoma Multiforme (TCGA, Provisional) database. The RPPA data acquired for our study can be accessed in the Supplementary Data File along with the TCGA IDH-wt glioma and GBM data used for analysis.

## Electronic supplementary material

Supplementary Information

Dataset
