# Supplementary material for: 5-Aminolevulinic Acid Guided Sampling of Glioblastoma Microenvironments Identifies Pro-Survival Signaling at Infiltrative Margins
Source: Sci Rep. 2017 Nov 15;7:15593. doi: 10.1038/s41598-017-15849-w (PMC5688093; doi:10.1038/s41598-017-15849-w)
Supplement: Supplementary file 1 — Supplementary Information [file 41598_2017_15849_MOESM1_ESM.pdf]

5-Aminolevulinic Acid Guided Sampling of Glioblastoma Microenvironments Identifies Pro-Survival Signaling at Infiltrative Margins

Supplemental Information

James L. Ross[1,4,7], Lee AD Cooper[2,5,6,7,8], Jun Kong[2,5,6], David Gutman[3,5,6], Merete Williams[1], Carol Tucker-Burden[1], Myles R. McCrary[6,8], Alexandros Bouras[9], Milota Kaluzova[4], William D. Dunn Jr.[2], Duc Duong[3], Constantinos G. Hadjipanayis[9*], Daniel J. Brat[1,2,5,6*]

[1]Departments of Pathology and Laboratory Medicine, [2]Biomedical Informatics, [3]Neurology, [4]Pediatrics, [5]Winship Cancer Institute, [6]Emory University School of Medicine, [7]Emory University Graduate Program in Cancer Biology, [8]Biomedical Engineering, Emory University / Georgia Institute of Technology, Atlanta, GA 30322.

[9]Department of Neurosurgery, Icahn School of Medicine at Mount Sinai, New York, NY 10003.

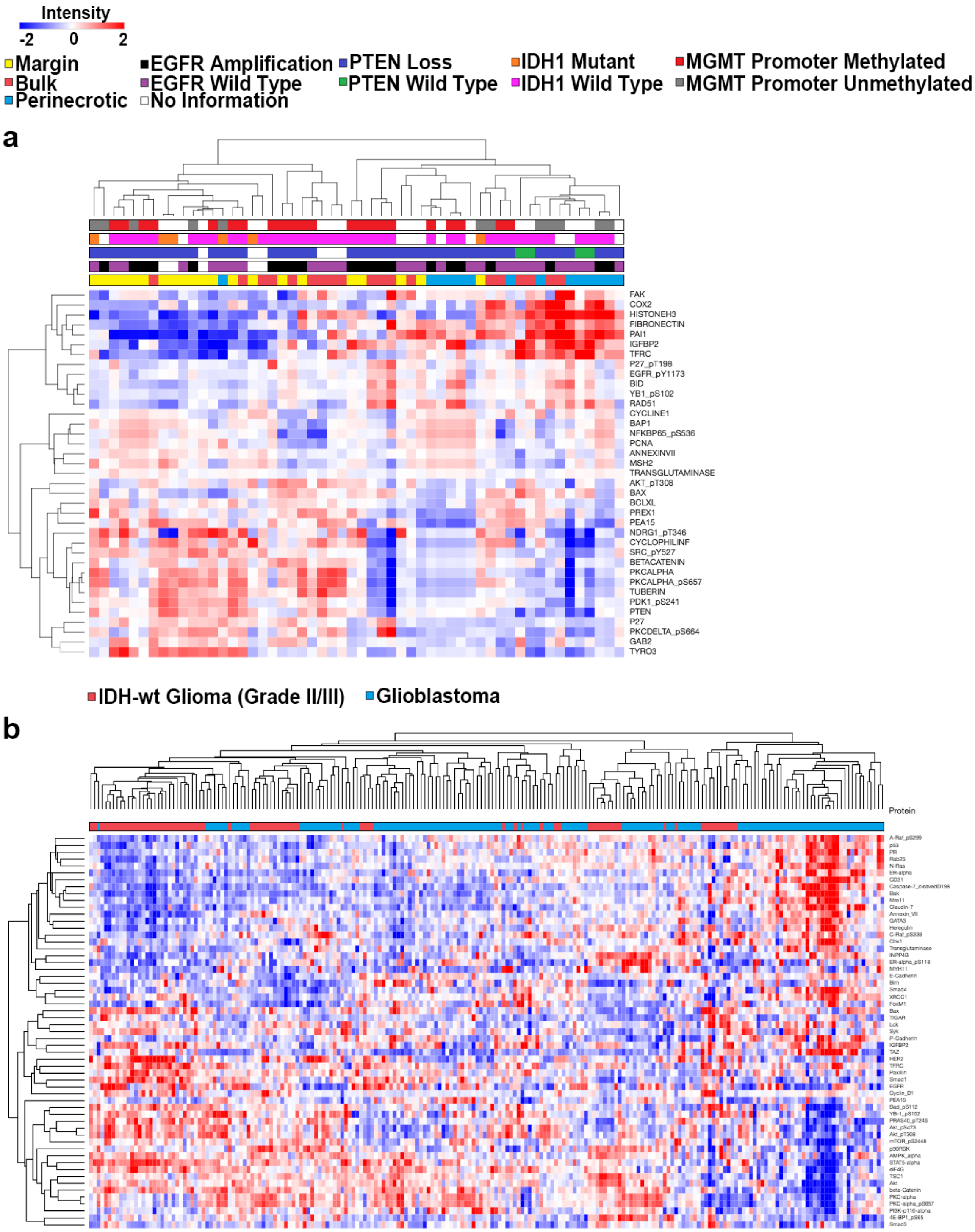

Supplemental Figure 1: (a) Unsupervised hierarchical clustering of the 37 differentially expressed proteins detected in the RPPA panel demonstrating that the tumor regions have distinct protein expression signatures. TM samples cluster together, PN samples cluster

together, and BT samples are found in both clusters. Molecular profiling of each sample is also provided**. (b)** Unsupervised hierarchical clustering of all differentially expressed proteins (n= 57, $p<0.05$) in the TCGA dataset displaying two main clusters where IDH-wt grade II/III glioma samples tend to cluster together and GBM samples tend to cluster together.

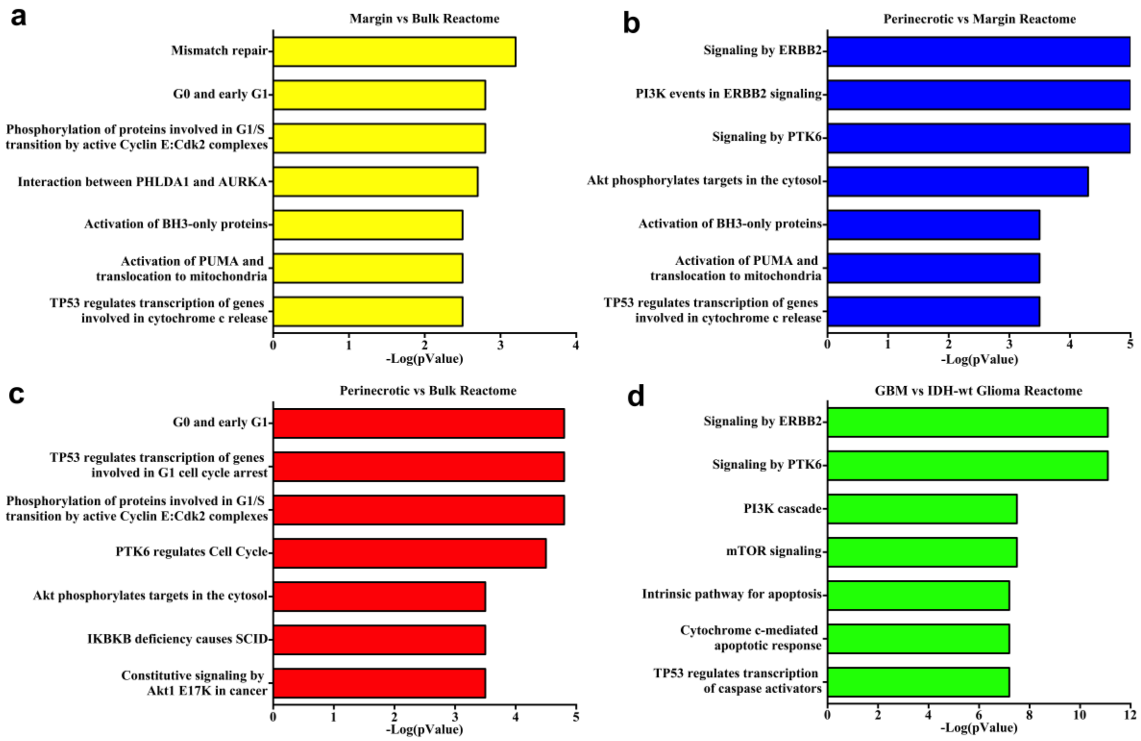

Supplemental Figure 2: (a-d) Reactome pathway analysis of the differentially expressed proteins for each pairwise comparison in our dataset and in the TCGA data. Data was generated using the ClueGO add-in for Cytoscape. The negative log of the Bonferroni corrected p-values was taken and plotted with the selected pathways using R. X-axis represents –Log(pValue) of the corresponding pathways on the y-axis.

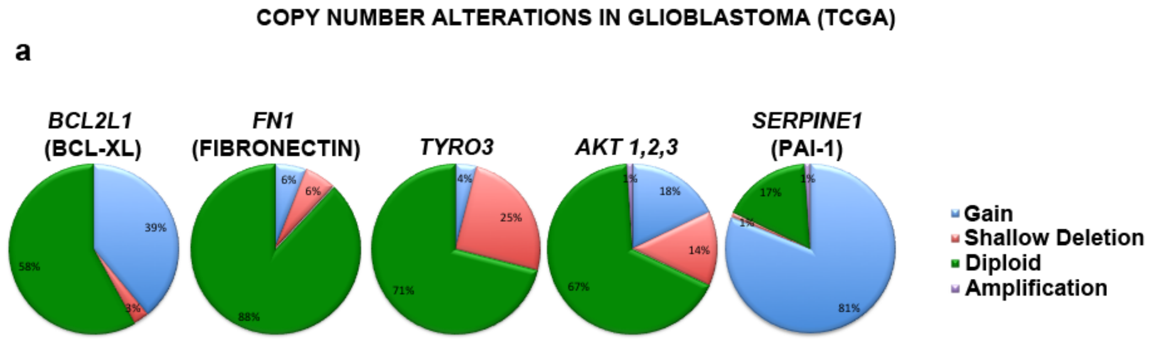

Supplemental Figure 3: (a) Copy number alterations for selected proteins used as validation in immunohistochemical and western blot analysis. Data is from the Glioblastoma Multiforme (TCGA, Provisional) database accessed on 20 June, 2017.

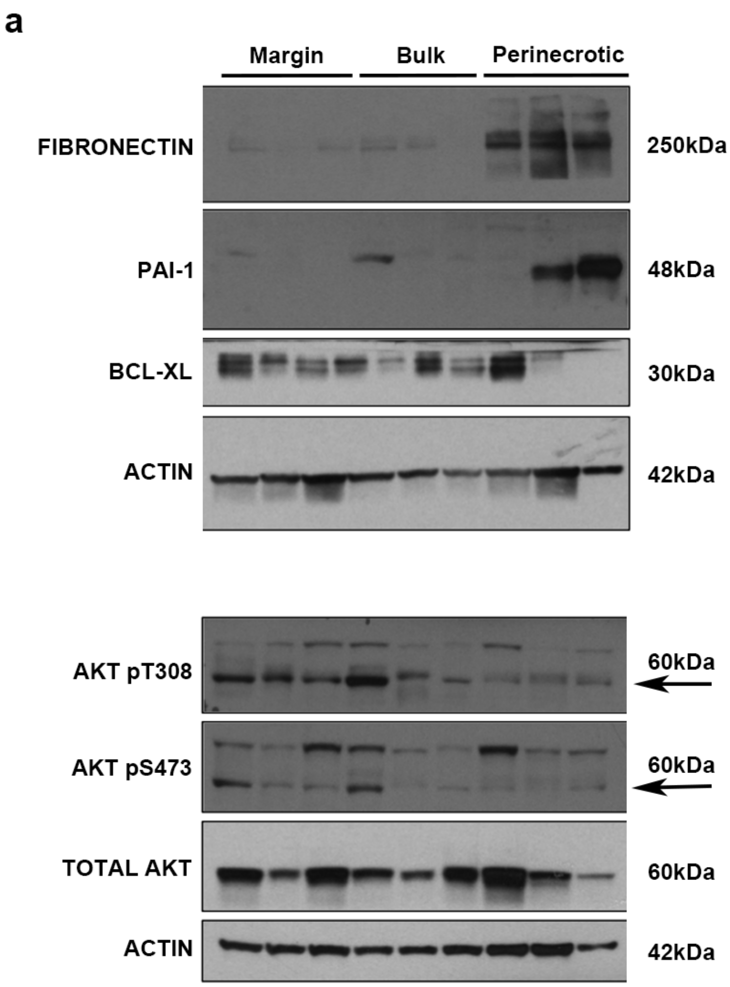

Supplemental Figure 4: (a) Uncropped full length western blots for Akt (pT308), Akt (pS473), Fibronectin, Bcl-XL, and PAI-1 using corresponding patient tissue samples. n=3 patients for each tumor region. Proteins were probed for on two separate blots.
